# Antibiotic Resistance in the Food Chain: A Developing Country-Perspective

**DOI:** 10.3389/fmicb.2016.01881

**Published:** 2016-11-23

**Authors:** Luria Leslie Founou, Raspail Carrel Founou, Sabiha Yusuf Essack

**Affiliations:** ^1^Antimicrobial Research Unit, Department of Pharmacy, School of Health Sciences, University of KwaZulu-NatalDurban, South Africa; ^2^Department of Microbiology, Centre of Expertise and Biological Diagnostic of CameroonYaoundé, Cameroon

**Keywords:** antibiotic resistance, zoonotic transmission, food chain, developing country, one health approach, global action plan, prevention, containment measures

## Abstract

Antibiotics are now “endangered species” facing extinction due to the worldwide emergence of antibiotic resistance (ABR). Food animals are considered as key reservoirs of antibiotic-resistant bacteria with the use of antibiotics in the food production industry having contributed to the actual global challenge of ABR. There are no geographic boundaries to impede the worldwide spread of ABR. If preventive and containment measures are not applied locally, nationally and regionally, the limited interventions in one country, continent and for instance, in the developing world, could compromise the efficacy and endanger ABR containment policies implemented in other parts of the world, the best-managed high-resource countries included. Multifaceted, comprehensive, and integrated measures complying with the One Health approach are imperative to ensure food safety and security, effectively combat infectious diseases, curb the emergence and spread of ABR, and preserve the efficacy of antibiotics for future generations. Countries should follow the World Health Organization, World Organization for Animal Health, and the Food and Agriculture Organization of the United Nations recommendations to implement national action plans encompassing human, (food) animal, and environmental sectors to improve policies, interventions and activities that address the prevention and containment of ABR from farm-to-fork. This review covers (i) the origin of antibiotic resistance, (ii) pathways by which bacteria spread to humans from farm-to-fork, (iii) differences in levels of antibiotic resistance between developed and developing countries, and (iv) prevention and containment measures of antibiotic resistance in the food chain.

## Introduction

Antibiotics are natural, synthetic, or semi-synthetic substances which interfere that the growth of or kill microorganisms, specifically bacteria, and are used to treat or prevent infections in humans and animals (O'Neill, 2015a; WHO, 2015a). Their development is one of the greatest accomplishments of modern medicine, their availability being vital to medical practices in communities and hospitals globally (O'Neill, 2015a; WHO, 2015a). Antibiotics are now an “endangered species” facing extinction due to the worldwide emergence of antibiotic resistance (ABR) and the void in the development of new therapeutic substances (O'Neill, 2015a; WHO, 2015a). An increasing number of treatment failures have been reported in patients with infections caused by multi-, extensive-, and pan-drug resistant bacteria. Once antibiotics normally used against bacteria are no longer effective, it becomes necessary to use others, so-called “reserve” or “last resort” options that are often more expensive and/or toxic preparations (O'Neill, 2015a).

Antibiotic use is the key factor in the selection of resistant bacteria, with community and hospital settings forming the principal ecological niches of emergence in human health (O'Neill, 2015a; WHO, 2015a). However, it is the use of antibiotics in animals that has contributed to the magnitude of the global challenge of ABR (FAO, 2015). In the intensification of food animal production and aquaculture, antibiotics are administered not only as therapy, but also as metaphylactics, where the identification of disease symptoms in one animal prompts the treatment for the whole flock or herd, and as prophylactics where, sub-therapeutic doses are administered to counteract the adverse effect of stress responses that generally lead to infectious diseases (FAO, 2015). In addition, the extensive use of antibiotics as growth promoters for the rapid growth of food animals and fish exacerbates the emergence and spread of ABR (FAO, 2015). The emergence of ABR in the food chain is considered a cross-sectoral problem, as (i) antibiotics are widely used in aquaculture, livestock production, and crop culture (Acar and Moulin, 2006; FAO, 2015), (ii) antibiotic-resistant bacteria and antibiotic-resistant genes (ARGs) can easily spread at each stage of the food production chain (da Costa et al., 2013; FAO, 2015), and (iii) can cause infections in humans (Chang et al., 2015; WHO, 2015a). The emergence of ABR along the food chain is thus a major global public health issue, with several studies having reported food animals and products being colonized and/or infected and contaminated by antibiotic-resistant strains, such as methicillin-resistant *Staphylococcus aureus* (MRSA) (Price et al., 2012), antibiotic-resistant *Campylobacter* spp. (Ewnetu and Mihret, 2010), and extended spectrum-beta-lactamase (ESBL) producing-*Enterobacteriaceae* (viz. *Salmonella* spp., *Shigella* spp., *Escherichia coli, Klebsiella* spp., etc.; Fischer et al., 2012; Al Bayssari et al., 2015). The situation has been recently worsened with the emergence of antibiotic-resistant bacteria having significant pandemic potential, such as carbapenem-resistant *Enterobacteriaceae* (CRE) (harboring a VIM-1 carbapenemase resistant to the beta-lactam antibiotics family plus additional co-resistance; Fischer et al., 2012) and colistin-resistant *E. coli* (harboring mcr-1 gene and co-resistance genes; Liu et al., 2016), as well as emerging livestock associated-methicillin resistant *S. aureus* (LA-MRSA; Price et al., 2012), in German, Chinese, and Dutch pigs, respectively. In fact, these resistant strains recognized as having an animal origin, are associated with multiple hosts adaptability (Ewers et al., 2012; Price et al., 2012; Fernandes et al., 2016; Liu et al., 2016), virulence mechanisms (Ewers et al., 2012; Price et al., 2012) and high genetic exchanges (Price et al., 2012; Liu et al., 2016), are serious threats to the world as they could lead to the emergence of new and more resistant, virulent and mobile strains, unknown from the human immune system (Wulf and Voss, 2008). This phenomenon termed “superburg” is one of the main concern feared by the international scientific community as it could result in pandemic situations caused by as resistant and virulent bacteria (Wulf and Voss, 2008; Ewers et al., 2012).

Antibiotic-resistant bacteria may reach humans (i) indirectly along the food chain through consumption of contaminated food or food derived products, and (ii) following direct contact with colonized/infected animals or biological substances such as blood, urine, feces, saliva, and semen among others (Chang et al., 2015). Given the direct interaction of humans with the animal-ecosystem interface, it is essential to prevent the zoonotic transmission of antibiotic-resistant bacteria and ARGs from food animals-associated reservoirs to humans. The Food and Agriculture Organization of the United Nations (FAO), the World Organization for Animal Health (OIE), and the World Health Organization (WHO) endorsed the One Health approach, affirming that healthy animals contribute to healthy people and environments (FAO, 2015; WHO, 2015a). In keeping with this, supranational programs and systems for monitoring antimicrobial resistance in animals and foodborne (viz. originating from food or food products) pathogens, namely, the Global Foodborne Infections Network, the WHO's Advisory Group on Integrated Surveillance of Antimicrobial Resistance and the Codex Alimentarius Commission, have been established. The WHO's Global Action Plan (WHO, 2015a) and FAO's Action Plan on Antimicrobial Resistance (FAO, 2016) were recently published to address this worldwide threat.

Several programs monitoring antibiotic use and ABR in food animals, foodstuff, and humans, have been successfully implemented in high resource settings such as the European Union (EU), Denmark, Netherlands, Sweden, Japan and United States (US) (The Japanese Veterinary Antimicrobial Resistance Monitoring System in the Field of Animal Hygiene (JVARM), 2013; DANMAP, 2014; FDA, 2014; NethMap-MARAN, 2015; SWEDRES-SVARM, 2015; EFSA and ECDC, 2016). A number of low- and middle-income countries (LMICs) have also initiated efforts to contain ABR, albeit with a focus on human health. India introduced and has begun to implement national guidelines for antibiotic use in 2013. China launched a National Antibiotic Restraining Policy to reduce antibiotic consumption in the country, Thailand implemented a national strategy for emerging diseases, including ABR, and a policy for rational drug use in 2011, as well as an ABR Containment Program for 2012–2016. South Africa developed and implemented a National Framework of Antimicrobial Resistance for 2014–2024.

The majority of LMICs are however still far behind high-resource settings in terms of curbing the spread of ABR generally, and via the food chain specifically. This despite that substantial risk factors for communicable diseases and extensive animal-occupational exposure exist in LMICs. The true burden of ABR in food animals is only partially documented and its threat via the food chain is under-estimated in low resource settings. Given the expansion of the human population, globalization of trade in animals and food products, international travels and host movements, ABR can easily spread globally via the food chain (Holmes et al., 2016). If preventive and containment measures are not applied locally, nationally, regionally and internationally, the limited interventions in one country or continent, for instance, in the developing world, can compromise the efficacy and endanger the policies of containment of ABR implemented in other parts of the world, the best-managed high-resource countries included. ABR is therefore a global problem, requiring integrated, multi-sectoral, and global solutions, as there are no geographic boundaries to its worldwide spread.

This review evidences the danger of ABR in the food chain and particularly in developing countries as a serious global public health threat. First, it assesses and summarizes background information on the emergence of ABR. Second it highlights transmission routes of ABR along the food chain; third, it compares the current status of ABR in food animals in developed vs. developing countries and finally, it delineates prevention and containment measures of ABR from farm-to-fork.

## Emergence of antibiotic resistance

Antibiotic use has always been associated with the development of resistance. Indeed, whenever an antibiotic is consumed, it eliminates susceptible bacterial cells, leaving behind or selecting those unusual strains that continue to grow in its presence through a Darwinian selection process. Those resistant variants then multiply, becoming the predominant bacterial population, and transmit their genetic resistance characteristics to offspring (Apata, 2009; Holmes et al., 2016). Such phenomenon can occur in saprophytic, commensal and pathogenic bacteria in humans, animals. and the environment.

**Figure 1:**
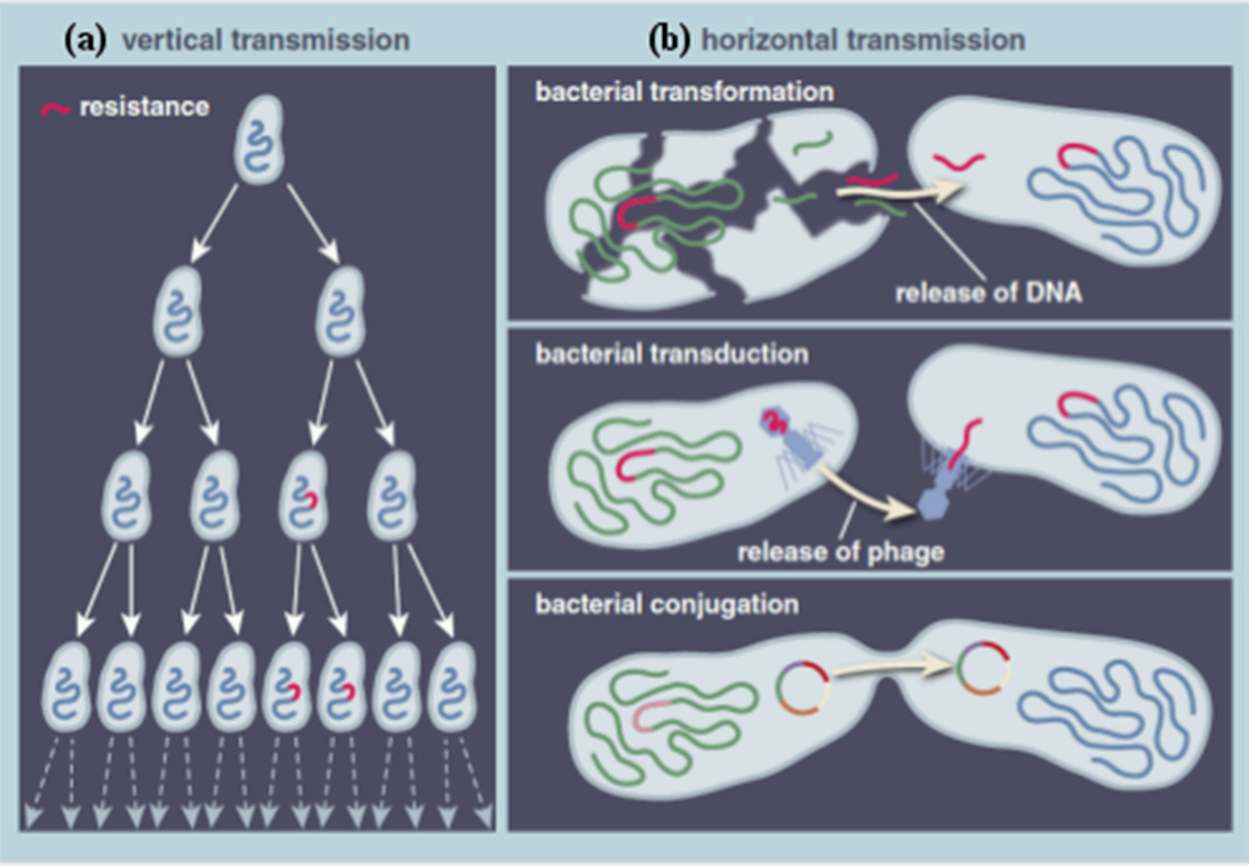
**Principal biological pathways involved in the evolution and development of antibiotic resistance. (A)** Vertical transmission **(B)** Horizontal transmission. Reprinted by permission from American Scientist (Dantas and Sommer, 2014), Copyright (2014).

Food animals, fish, and vegetables are considered large reservoirs of antibiotic-resistant bacteria, as the food production chain is an ecosystem composed of different ecological niches, where large quantities of antibiotics are used and numerous bacteria co-exist (Acar and Moulin, 2006). There are two principal biological pathways involved in the evolution and development of ABR. First, resistance can be mediated by a pre-existing phenotype in natural bacterial populations. During the evolutionary process, bacterial cells accumulate genetic errors in existing genes (in the chromosome or plasmid) and transfer the resistant genes to progeny cells via vertical gene transfer (VGT), leading to an *innate* or *intrinsic* or *natural* resistance (Figure 1). The second scenario, called *acquired* resistance, involves genetic exchanges within and between bacterial species (Apata, 2009; Holmes et al., 2016). It implies horizontal gene transfer (HGT) and the acquisition of new resistant genes harbored on mobile genetic elements, such as plasmids, integrons, transposons, insertion sequences, and phage-related elements. Such genetic materials are transferred through conjugation (transfer of DNA from donor to recipient bacterial through cell-to-cell contact and aided by a fertility-factor called pili), transformation (naked-DNA present in the environment is taken-up by the recipient cell), and transduction (a bacteriophage acts as vector and inserts DNA into recipient cell; Apata, 2009; Holmes et al., 2016; Figure 1).

HGT leads to new bacterial populations carrying a combination of new resistant genes and mechanisms, resulting in different resistance profiles (Acar and Moulin, 2006). This genetic process has been identified as the main driving force behind the spread of ABR, as it allows interspecies transmission, which is especially effective in the bacterial microbiome (gastro-intestinal tract, naso-pharyngeal mucosa, and skin) of animals and humans (Apata, 2009; Holmes et al., 2016). It is further important to note that VGT and HGT may occur concomitantly in a bacterial population (Acar and Moulin, 2006). These two types of mechanisms create a resistant population where selective pressure exerted by antibiotic use will substantially increase their number and risk of spread (Apata, 2009; Holmes et al., 2016). Antibiotic-resistant bacteria may act as reservoir of ARGs for other bacteria, which might be conserved within the bacterial population, even without the related antibiotics (Apata, 2009; Holmes et al., 2016).

## Transmission routes of antibiotic resistance along the food chain

The spread of ABR is possible along the food chain through direct or indirect contact. Direct contact occurs following immediate exposure of humans with animals and biological substances (such as blood, urine, feces, milk, saliva, and semen), and enhances the rapid and easy dissemination of resistant bacteria from host-to-host. Occupationally exposed workers such as veterinarians, farmers, abattoir workers and food handlers, as well as those directly in contact with them, are at high risk of being colonized or infected with antibiotic-resistant bacteria (Marshall and Levy, 2011). Although this transmission did not initially appear as a threat at population-health level, it is now acknowledged that exposed workers and their families provide a likely route for the entry of antibiotic-resistant bacteria and ARGs into the community and health care settings, where subsequent exchanges and the acquisition of resistance mechanisms are evident (Marshall and Levy, 2011).

**Figure 2:**
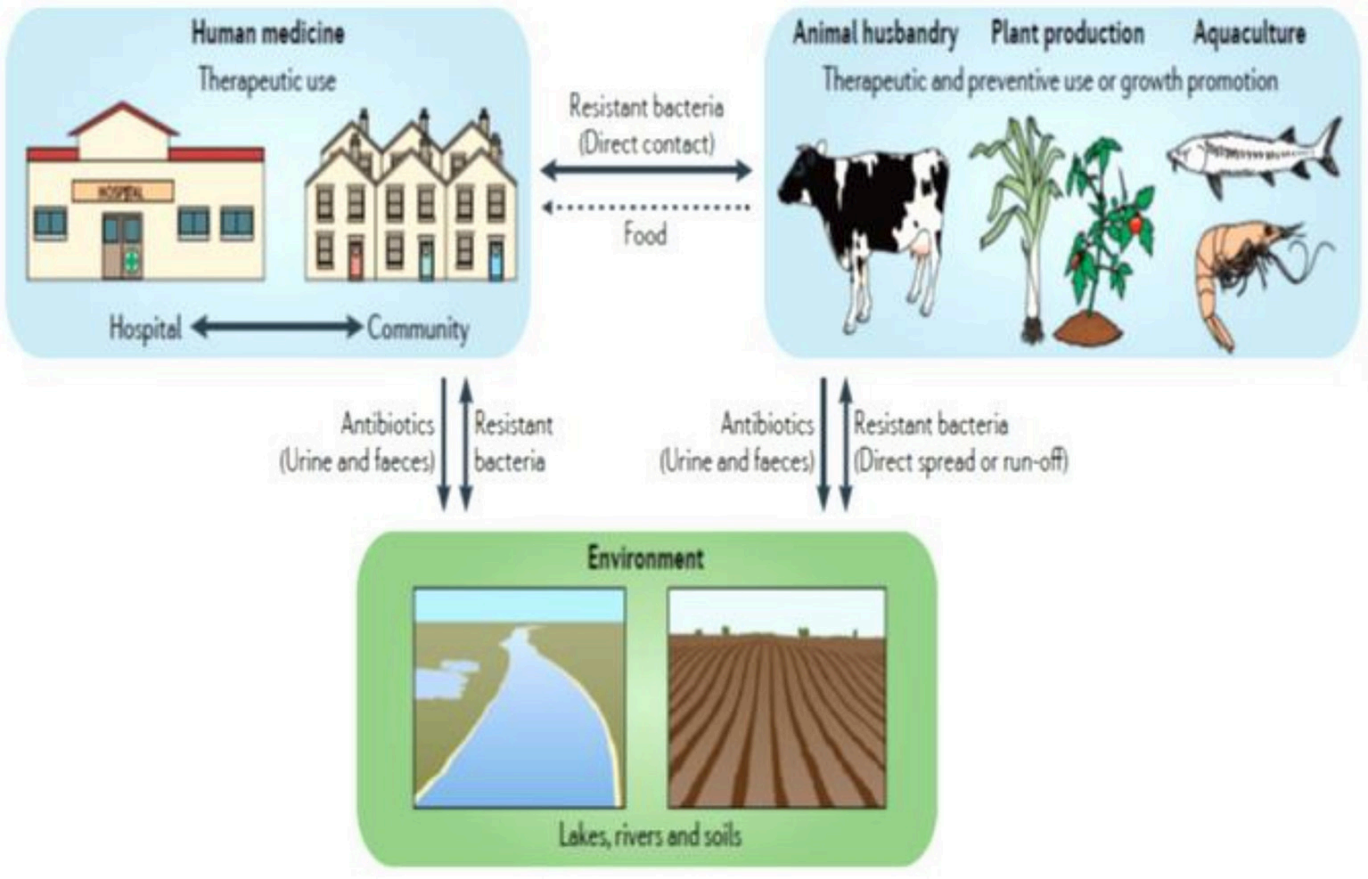
**Antibiotic use and different transmission routes of antibiotic resistance in the food chain**. Adapted by permission from MacMillan Publishers Ltd: [Nature Reviews Microbiology], (Andersson and Hughes, 2014), Copyright (2014).

In addition, the human population may be exposed indirectly to antibiotic-resistant bacteria and ARGs via contact with or consumption of contaminated food products (e.g., meat, eggs, milk, and dairy products). This indirect transmission through the food chain is a far-reaching and more complex pathway (Figure 2). Recently, numerous reports have described the presence of large quantities of antibiotic-resistant bacteria and ARGs in various food products (ready-to-eat meat, cooked meat, and bulk milk) from various animal sources, such as cattle, poultry, swine, goat, and sheep, and from different stages of food production (Price et al., 2012; Coetzee et al., 2016; Liu et al., 2016). Several studies have further identified similar or clonally related antibiotic-resistant bacteria and ARGs of animal origin in human populations without occupational exposure, providing likely evidence for transfer following the consumption and/or handling of food (Acar and Moulin, 2006; Marshall and Levy, 2011). Farmers, abattoir workers and food handlers as well as consumers are thus the large number of people directly at risk of acquiring antibiotic-resistant bacteria via the food chain. In the developing world, where biosecurity and food safety measures are limited along the farm-to-fork continuum and where humans interact intimately with animals and the environment, the public health risk is likely to be associated with both the direct and indirect transmission of antibiotic-resistant bacteria and ARGs (Padungtod et al., 2008). In contrast, in developed countries, the indirect contamination seems to be more prevalent as antibiotic-resistant bacteria and ARGs emerging on-farms, are maintained throughout the food production and contaminated food products reach the end consumers to create foodborne infections (EFSA and ECDC, 2015).

A large proportion of antibiotics are not transformed into inactive compounds, and retain their activities after renal or biliary excretion (Thanner et al., 2016). The dissemination of active antibiotics, metabolites or degradation products of those antibiotics, termed antibiotic residues, as well as ARGs and antibiotic-resistant bacteria excreted via food animals waste, have established the environment as another important reservoir of ABR (da Costa et al., 2013; Zhu et al., 2013; Woolhouse et al., 2015; Thanner et al., 2016; Figure 2). Farm soils, manure, and wastewater have been identified as “hot spots” of ABR pollution, with antibiotic residues being detected across the world (Zhu et al., 2013; Wu et al., 2014; Thanner et al., 2016). Antibiotic residues lead to various adverse effects for human health, such as allergic hypersensitivity reactions, toxic effects, hepatotoxicity, nephropathy, mutagenicity, carcinogenicity, and ABR (Mensah et al., 2014). Although the concern associated with the presence of antibiotic residues in food animals has been mitigated in high resource economies, with <1% of these substances detected in food products in the European Union (EU), it remains a major public health threat in LMICs, with prevalence ranging from 4 to 90% (Mensah et al., 2014). The high prevalence of these residues in various ecological niches in the farm-to-fork continuum exacerbates the issue of ABR in the developing world, as it enhances the pool of antibiotic-resistant bacteria and ARGs in the ecosystem as a result of exposure to sub-inhibitory concentrations of antibiotics and their residues.

HGT has further been documented in environmental niches, such as manure, water and soil, leading to microbial communities containing various levels of antibiotic-resistant bacteria and ARGs (Zhu et al., 2013; Thanner et al., 2016). Several LMICs use animal and human waste, without appropriate treatment, as fertilizer in crop lands and for feeding of fish and shellfish in aquaculture (Zhu et al., 2013). The environmental pollution may therefore lead to the emergence and spread of new antibiotic-resistant bacteria and ARGs in food products, fish, shellfish, vegetables, feed and water, which may in turn colonize or infect animals and humans, thereby enhancing the public health risks associated with the presence of ABR in the food chain (Acar and Moulin, 2006; Thanner et al., 2016). The key issues regarding these reservoirs are (i) identifying the different ecological niches and pathways associated with the presence of ABR in the food chain, and, (ii) understanding the public health implications for the human population when the transfer of these antibiotic-resistant bacteria and ARGs from these intermingled reservoirs occurs. It further reveals knowledge gaps regarding the magnitude and dynamic nature of the spread of antibiotic-resistant bacteria and/or ARGs within and between different ecological niches in the farm-to-fork continuum, which deserves to be considered when assessing the transmission of ABR along the food chain (Acar and Moulin, 2006; Woolhouse et al., 2015). Substantial metagenomics studies ascertaining the bacterial resistome, the HGT between antibiotic-resistant bacteria present in the above niches, as well as providing phylogenetic evidence and transmission dynamics within and between populations, are vital to determine the evolutionary origins of bacterial lineages, and may provide new perspectives on understanding the implications of ABR in the food chain (Woolhouse et al., 2015).

## Current status of antibiotic resistance in the food chain

Different types of antibiotic-resistant bacteria have increasingly been identified in food animals, products, and feeds as well as in humans (exposed or not). Such antibiotic-resistant bacteria are either (i) indicator or commensal, (ii) foodborne, or (iii) emergent organisms. ARGs emerging in commensal bacteria are likely spread to foodborne ones and vice versa, while the numerous genetic exchanges occurring between different ecological niches lead to new resistant bacteria or genes or combination thereof. Antibiotic-resistant bacteria and ARGs of animal origin are globally reported, with the prevalence differing greatly between geographic locations and differentially resourced settings. MRSA and ESBL-producing *Enterobacteriaceae* (e.g., *Salmonella* spp., *E. coli, Shigella* spp., *K. pneumoniae, Enterobacter* spp., etc.) are among the best-known examples.

### Developed countries

In high-resource developed countries, an assortment of systems and programs to monitor antibiotic use, as well as ABR in food animals, food products, and humans have been implemented (Table 1). Such initiatives have led to the substantial decrease of antibiotic consumption and rates of resistance in these settings. In Norway for instance, The NORM/NORM-VET reported a very low annual antibiotic use of 5.927 tons in terrestrial animals in 2014, representing a total decrease of 38% from 1995 to 2014 (NORM/NORM-VET, 2015). The 2014's DANMAP report revealed a decrease of 2% compare to 2013, with 114 tons of active compounds consumed in livestock (DANMAP, 2014). Similarly, in the Netherlands, 207 tons of antibiotics were consumed in 2014, showing a reduction of 4.4% from 2013, and an overall decrease of 58.1% over the period 2009–2014 (NethMap-MARAN, 2015). In the European Union (EU), antibiotics were banned for growth-promotion in 2006, while the United States (US) implemented legislation based on a voluntary cessation of antibiotic use for growth-promotion as well as re-labeling of antibiotics.

**Table 1:** **Examples of programs for surveillance and containment of antibiotic resistance**.

**Monitoring programs**	**Country**	**Target population**	**Target bacteria**	**References**
Danish Integrated Antimicrobial Resistance Monitoring and Research Programme (DANMAP)	Denmark	Humans, animals, and food products	*Salmonella* spp.	DANMAP, 2014
			*Campylobacter* spp.	
			*Enterococcus* spp.	
			*Escherichia coli*	
			*Klebsiella pneumonia*	
			*P. aeruginosa*	
			*Streptococcus* spp.	
			*Staphylococcus aureus*	
Norwegian Surveillance System for Antimicrobial Drug Resistance (NORM/NORM-VET)	Norway	Humans, animals, and food products	*Escherichia coli*	NORM/NORM-VET, 2015
			*Enterococcus* spp.	
			*Salmonella* spp.	
			*Campylobacter* spp.	
			*Yersinia enterocolitica*	
			*Shigella* spp.	
Swedish Veterinary Antimicrobial Resistance Monitoring (SVARM)	Sweden	Animals and food products	*Salmonella* spp.	SWEDRES-SVARM, 2015
			*Campylobacter* spp.	
			*Staphylococcus aureus*	
			*Staphylococcus pseudointermidius*	
			*Enterobacteriaceae*	
			*Enterococcus* spp.	
European Antimicrobial Resistance Surveillance Network (EARS-Net)	Multinational^a^	Humans	*Streptococcus pneumoniae*	ECDC, 2015
			*Staphylococcus aureus*	
			*Enterococcus* spp.	
			*Escherichia coli*	
			*Klebsiella pneumoniae*	
			*P. aeruginosa*	
European Surveillance of Antimicrobial Consumption Network (ESAC-Net)	Multinational^b^	Humans	*Streptococcus pneumoniae*	ECDC, 2014
			*Staphylococcus aureus*	
			*Enterococcus* spp.	
			*Escherichia coli*	
			*Klebsiella pneumoniae*	
			*P. aeruginosa*	
Monitoring and analysis of food-borne diseases in Europe (EFSA)	Multinational^c^	Humans, animals and food products	*Salmonella* spp.	EFSA and ECDC, 2016
			*Campylobacter* spp.	
			*Escherichia coli*	
			*Staphylococcus aureus*	
Monitoring of Antimicrobial Resistance and Antibiotic Usage in Animals in the Netherlands (MARAN)	Netherlands	Animals and food products	*Salmonella* spp.	NethMap-MARAN, 2015
			*Campylobacter* spp.	
			*Escherichia coli*	
			*Enterococcus* spp.	
National Antimicrobial Resistance Monitoring System (NARMS)	United States	Humans, animals and food products	*Salmonella* spp.	FDA, 2014
			*Campylobacter* spp.	
			*Enterococcus* spp.	
			*Shigella spp*.	
			*Escherichia coli*	
			*Vibrio spp*. other than *V. cholera*	
Canadian Integrated Program for Antimicrobial Resistance Surveillance (CIPARS)	Canada	Humans, animals and food products	*Salmonella* spp.	Government of Canada, 2014
			*Campylobacter* spp.	
			*Enterococcus* spp.	
			*Escherichia coli*	
L'Observatoire National de l'Epidémiologie de la Résistance Bactérienne aux Antibiotiques (ONERBA)	France	Humans and Animals	*Enterobacteriaceae and non-fermenters*	ONERBA, 2015
			*Staphylococcus aureus*	
			*Streptococcus* spp.	
			*Enterococcus* spp.	
The Japanese Veterinary Antimicrobial Resistance Monitoring System in the Field of Animal Hygiene (JVARM)	Japan	Animals	*Salmonella* spp.	The Japanese Veterinary Antimicrobial Resistance Monitoring System in the Field of Animal Hygiene (JVARM), 2013
			*Campylobacter* spp.	
			*Enterococcus* spp.	
			*Escherichia coli*	
Japanese Nosocomial Infections Surveillance (JANIS)	Japan	Humans	*Enterobacteriaceae and non-fermenters*	JANIS, 2016
			*Staphylococcus* spp.	
			*Streptococcus* spp.	
			*Enterococcus* spp.	
The Finnish Veterinary Antimicrobial Resistance Monitoring and Consumption of Antimicrobial Agents report (FINRES-VET)	Finland	Animals and food products	*Escherichia coli*	FINRES-Vet, 2007-2009
			*Enterococcus* spp.	
			*Salmonella* spp.	
			*Campylobacter* spp.	
			*Staphylococcus aureus*	
			*Staphylococcus pseudointermidius*	
Pilot Surveillance Program for Antimicrobial Resistance in Bacteria of Animal Origin	Australia	Animals	*Campylobacter* spp.	The department, 2007
			*Escherichia coli*	
			*Enterococcus* spp.	
Colombian Integrated Program for Antimicrobial Resistance Surveillance (COIPARS)	Colombia	Humans, Food animals, and products	*Salmonella* spp.	COIPARS, 2011
			*Campylobacter* spp.	
			*Escherichia coli*	
Pilot Integrated Food Chain Surveillance System	Mexico	food animals and products	*Salmonella* spp.	Zaidi et al., 2008, 2012
			*Campylobacter* spp.	

a *33 European countries*.

b *34 European countries*.

c *28 European countries*.

Transmission of antibiotic-resistant bacteria via direct contact from animals to exposed workers, and indirectly following consumption of contaminated food products (broiler meat, beef, pork, milk, etc.), is well-documented in these countries (Table 2; DANMAP, 2014; NORM/NORM-VET, 2015; EFSA and ECDC, 2016). In the EU, a new legislation on monitoring of ABR in animals and food was implemented to ensure the comparison of similar and high-quality data in 2013 (EFSA and ECDC, 2016). As a result, harmonized data from the 28 EU member states were collated by the EFSA and ECDC to provide continent-specific estimates of ABR in food animals and products. The 2014's EFSA/ECDC EU Summary report on ABR in zoonotic bacteria revealed increased prevalence of multi-drug resistant (MDR) *S. Infantis* (more than 70% in broiler meats), MDR-*E. coli* (55% in broiler meats) and MRSA (26.5% in all food animals) in the EU (EFSA and ECDC, 2016). These bacteria are recognized as a serious public health threat due to their resistance to several antibiotics, often critically important for human health, thereby limiting the choice of effective antibiotic agents available for treatment.

**Table 2:** **Prevalence of antibiotic-resistant bacteria isolated from food animals and products in developed and developing countries**.

**Country**	**Isolation year**	**Origin/type of specimens**	**Antibiotic-resistant bacteria**	**Prevalence (%)**	**References**
**DEVELOPED COUNTRIES**					
European Union	2014	Broiler meat	MDR-*Salmonella Infantis*	>70	EFSA and ECDC, 2016
		Broiler meat	MDR-*E. coli*	55	
Norway	2014	Broiler	ESBL-producing *E. coli*	36	NORM/NORM-VET, 2015
		Broiler meat	ESBL-producing *E. coli*	30	
		Broiler	Vancomycin resistant *Enterococcus* spp.	7	
Netherlands	2014	Poultry	ESBL-producing *Salmonella* spp.	12	NethMap-MARAN, 2015
		Poultry	Fluoroquinolone-resistant *Salmonella* spp.	43	
		Pig	ESBL/AmpC-*E.coli*	18	
		Dairy cow	ESBL/AmpC-*E.coli*	9	
		Turkey meat	ESBL/AmpC-*E.coli*	51	
		Poultry meat	ESBL/AmpC-*E.coli*	67	
Denmark	2014	Pigs	MDR-*Salmonella* spp.	7	DANMAP, 2014
		Broiler meat	ESBL-producing *E. coli*	9	
United States	2012–2013	Turkey	MDR-*E. coli*	62	FDA, 2014
		Turkey	MDR-non-typhoidal *Salmonella*	34	
		Chicken	MDR-*E. coli*	62	
		Pig	MDR-*E. coli*	22	
		Ground beef	MDR-non-typhoidal *Salmonella*	20	
**DEVELOPING COUNTRIES**					
China	2015	Pig	Plasmid mediated colistin resistant *E. coli*	21	Liu et al., 2016
		Raw meat	Plasmid mediated colistin resistant *E. coli*	15	
Thailand	2007	Poultry	ESBL-producing *S. Typhimurium*	77.3	Padungtod et al., 2008
		Pig	ESBL-producing *S. Typhimurium*	40.4	
	2012–2013	Pig	ESBL-producing *E. coli*	77	Boonyasiri et al., 2014
		Pork	ESBL-producing *E. coli*	61	
		Pork	MDR-*A. baumannii* and *P. aeruginosa*	40	
		Poultry	ESBL-producing *E. coli*	40	
		Poultry meat	ESBL-producing *E. coli*	50	
Vietnam	2007	Poultry	MDR-*E. coli*	91.5	Usui et al., 2014
Indonesia	2007	Poultry	MDR-*Enterococcus faecalis*	84.5		
Brazil	2000–2016	Pigs	Plasmid mediated colistin resistant *E. coli*	1.8	Fernandes et al., 2016
		Chicken	Plasmid mediated colistin resistant *E. coli*	5	
Egypt	2010	Meat and dairy products	MDR-Shiga-toxin-producing *E. coli* O157:H7	57.4	Ahmed and Shimamoto, 2015a
	2010	Meat and dairy products	MDR-*Shigella spp*.	89	Ahmed and Shimamoto, 2015b
Algeria	2005–2006	Broilers	MDR- *Salmonella spp*.	51	Elgroud et al., 2009
Tunisia	2010–2011	Raw meat	MDR-*Enterococcus spp*.	24.5	Klibi et al., 2013
		Raw meat	LA-MRSA ST398	0.6	Chairat et al., 2015
South Africa	2014	Cattle	MDR-*E. coli*	100	Iweriebor et al., 2015
	2015	Poultry	Plasmid mediated colistin-resistant *E. coli*	79	Coetzee et al., 2016

*MDR, Multi-drug resistant*.

In a country-specific context, the NORM/NORM-VET detected high levels of ESBL-producing *E. coli* from broilers (36%) and broiler meat samples (30%) in Norway, whereas rates of vancomycin resistant *Enterococcus* spp. were quite low (7%) in 2014 (NORM/NORM-VET, 2015). Meanwhile, monitoring of ABR in food animals in the Netherlands by MARAN revealed a 12% prevalence of ESBL-producing *Salmonella* spp. and 43% of fluoroquinolone-resistant *Salmonella* spp. in poultry. It also showed diverse levels of ESBL/AmpC- producing *E. coli* in food animals and products, these being 67% in broilers, 18% in pigs, 9% in dairy cows, 51% in turkey meat, and 67% in poultry meat (NethMap-MARAN, 2015). The DANMAP reported low rates of MDR-*Salmonella* spp. (7%) in pigs and ESBL-producing *E. coli* (9%) in broiler meat in Denmark, confirming the country as an example in terms of containing ABR (DANMAP, 2014). Transmission of antibiotic-resistant bacteria along the food chain has nevertheless been demonstrated in the country, with a 28% prevalence of ESBL-producing *E. coli* from imported broiler meat samples (DANMAP, 2014). This evidence strongly suggests that there are no geographic borders to curtail the spread of antibiotic-resistant bacteria, and that their emergence in the food chain threatens the world equally. In US, the epidemiology of ABR in food animals appears to be notably different than in European countries, where the substantial ABR research has been carried out and antibiotic use for growth promotion has been prohibited for more than a decade. Antibiotic use in food animals is estimated to account 70% of the country's annual antibiotic consumption (FDA, 2014). The report from the NARMS, over the period 2012–2013, identified MDR-*E. coli* in turkey (62%), chicken (62%), and swine (22%) as well as MDR-non-typhoidal *Salmonella* in turkey (34%) and ground beef (20%). Several studies have further reported human colonization and outbreak situations associated with antibiotic-resistant bacteria and ARGs of food animal origins (FDA, 2014).

Notwithstanding the difference in rates of ABR demonstrated in the food chain in developed countries, it is important to mention that most have national programs to monitor antibiotic use and ABR from humans, animals, and food products (Table 1). In response to the data generated from these programs, these countries have adopted a set of policies and control measures to contain ABR while preserving their productivity. Denmark has taken major initiatives to address the challenge of ABR over the last 20 years, and actions taken by the Danish government have been acknowledged as a worldwide model (O'Neill, 2015b). The DANMAP, a system for monitoring the impact of withdrawal of antibiotic growth-promoters on the development of ABR in food animals and humans was established in the country to measure the effect of interventions and inform future policies (DANMAP, 2014). From the ban of avoparcin in 1995 up to 2008, poultry production has increased slightly and overall antibiotic consumption decreased by 90%, from 5 to 0.5 tons (used exclusively for therapy) in 2008 (DANMAP, 2014). The prevalence of vancomycin resistant *Enterococcus faecium* substantially declined in pig and poultry production between 1996 and 2008 (DANMAP, 2014). Several developed countries have also recently responded to and followed the recommendations of the WHO, OIE, and FAO tripartite alliance to strengthen the containment of ABR, with the development and implementation of their national action plans.

### Developing countries

In contrast with developed countries, the majority of LMICs have minimal or no programs or systems to monitor antibiotic use or ABR in food animals, food products, and humans, its true burden being only partially documented, and reliant on point-prevalence rather than long-term studies. Moreover, antibiotics are extensively used in agriculture for growth-promotion, and the danger of ABR in the food chain is further neglected and under-estimated (Eagar et al., 2012; Van Boeckel et al., 2014). For example, two-third of the 1500 tons of antibiotics sold for animal use over a 3 years' period (2002–2004) in South Africa were for growth-promotion purposes and included WHO-banned substances (Eagar et al., 2012). A recent modeling study suggests that up to a third of the global increase (67%) in antibiotic consumption in food animals, over the period 2010-2030, will be attributable to LMICs (Van Boeckel et al., 2015). For Brazil, Russia, India, China, and South Africa, this increase is projected to reach 99%, up to seven times the estimated population growth of these countries (Van Boeckel et al., 2015). By 2030, antibiotic use in food animals is estimated to be 51,851 tons in Asia alone, representing more than 80% of the global antibiotic consumption in food animals in 2010 (Van Boeckel et al., 2015). In 2007, China was the largest antibiotic producer and consumer, with 210,000 tons produced, with 46% being used in livestock production. The country was also the world's pig and poultry producer in 2014, with 56.7 and 17.5 million tons, respectively (Liu et al., 2016). The increase in agriculture and food production in LMICs parallels what developed countries experienced over the last 100 years when income rose (Collignon and Voss, 2015). The damaging consequences of this intensification in food production has resulted in several sub-optimal practices, such as over-crowding of animals, large quantities of antibiotics consumed, and often poor hygienic measures and farming practices (Collignon and Voss, 2015). The incidence of ABR is thus undoubtedly worse in developing countries, where humans interact intimately with animals and the environment, infectious diseases rates are higher, regulations on antibiotic use, and the development, implementation, and monitoring of ABR prevention and containment measures are rare and frequently non-existent.

Several studies have documented new resistance mechanisms and elevated rates of antibiotic-resistant bacteria in Asian and African developing nations (Table 2). A recent Chinese routine surveillance study revealed a highly mobile and transferable plasmid-mediated colistin-resistance gene, called mcr-1, in commensal *E. coli* isolated from pigs (21%) and raw meat (15%; (Liu et al., 2016)). The study also revealed that 1% of clinical isolates from hospitalized Chinese patients harbored this new resistant gene (Liu et al., 2016). It is thought that this gene originated from animals and spread into humans via the food chain (Liu et al., 2016). Plasmid mediated mcr-1 colistin-resistant *E. coli* were also detected in chicken (14/515) and pigs (2/515) in Brazil (Fernandes et al., 2016). This finding is of great concern, as although not frequently used in human medicine, colistin remains a drug of last resort, with the global emergence of extensively-drug resistant Gram negative bacteria.

In Thailand, high levels of ESBL-producing *Salmonella Typhimurium* in poultry (77.3%) and pigs (40.4%) were reported, with an overall 86% prevalence of MDR (Padungtod et al., 2008). In the same country, Boonyasiri et al. (2014) identified elevated prevalence of ESBL-producing *E. coli* in the food chain during the years 2012–2013, these being 77% in pigs, 40% in poultry, 77% in farmers, 76% in food handlers, 61% in pork, and 50% in chicken. The same study further reported 40% of MDR-*Acinetobacter baumannii* and *Pseudomonas aeruginosa* in pork (Boonyasiri et al., 2014). MDR-*E. coli* strains were also detected in chicken fecal samples in Vietnam (91.5%) and Indonesia (62.8%) with isolates being concomitantly resistant to up to 10 antibiotics (Usui et al., 2014). In Africa, there is a paucity of research and consequently data on antibiotic-resistant bacteria and ARGs in food animals and food products. However, high level of MDR-bacteria isolated from various food animals have been documented in several African countries. Ahmed and Shimamoto (2015a,b) reported MDR-Shiga-toxin-producing *E. coli* O157:H7 (57.4%) and MDR-*Shigella* spp. (89%) from food products (meat and dairy products) in Egypt, while a maximum prevalence (100%) of MDR-*E. coli* has been identified in rectal samples of cattle in South Africa (Iweriebor et al., 2015). MDR-*Enterococcus* spp. (24.5%) (Klibi et al., 2013) and MDR-*Salmonella spp*. (51%) (Elgroud et al., 2009) were isolated from meat samples and broilers in Tunisia and Algeria, respectively. A recent nation-wide surveillance in poultry settings described substantial increases in colistin-resistance in *E. coli* strains in 2015 in South Africa. Accordingly, the emergence of mcr-1 was confirmed in 79% of colistin-resistant strains isolated (Coetzee et al., 2016). Clinical isolates of colistin-resistant *E. coli* from hospitalized (*n* = 3) and outpatients (*n* = 6) were further detected in the same study (Coetzee et al., 2016). Carbapenemase producing-*P. aeruginosa* and *A. baumannii* have further been recently identified in food animals in Lebanon, confirming the serious threat associated with the emergence of ABR in the food chain (Al Bayssari et al., 2015).

The majority of developing countries are substantive exporters of food animals and food products. Brazil is the world's largest exporter of chicken meat (Fernandes et al., 2016) and Thailand exports not <70% of its poultry production (Boonyasiri et al., 2014). This means that antibiotic-resistant bacteria and/or ARGs emerging in these countries and other LMICs will easily spread across the world via the food chain (Boonyasiri et al., 2014; Fernandes et al., 2016; Holmes et al., 2016). Grami et al. (2016) recently reported high prevalence of plasmid mediated mcr-1 *E. coli* in fecal samples of chicken imported from European countries in Tunisia. This is also of grave concern as antibiotic-resistant bacteria and ARGs emerging in food animals and products in developed nations could endanger safety, public health in already overburdened health care settings of developing countries, where prevention and containment of ABR are limited and where high levels of ABR prevail (Table 2). This confirms that globalization of trade in food animals and products are major determinants of the worldwide dissemination of ABR from farm-to-fork.

Additionally, travelers visiting developing countries from developed nations may be colonized or infected by antibiotic-resistant bacteria and/or ARGs, and in turn become vectors of transmission in their home countries. Dutch travelers to North Africa (Tunisia), South America (Peru, Bolivia, and Colombia), and Asia (Thailand, China, Vietnam, Laos, and Cambodia) were recently reported to be colonized by colistin-resistant *E. coli* strains harboring mcr-1 gene (Coetzee et al., 2016). Likewise, the prevalence of ESBL-producing *Enterobacteriaceae*, colonization in Swedish, Dutch, and Australian tourists, increased from 2.4–8.6 to 30–49% following travel to India, Southeast Asia, and China (Boonyasiri et al., 2014). Travelers from developed nations seeking healthcare in LMICs, generally to avoid high costs, long delays or due to legal or cultural restrictions in their home countries, have also been involved in the spread of ABR (Chen and Wilson, 2013). A recent study confirmed that recent international travel was an independent risk factor for septicemia after trans-rectal prostate biopsy (Chen and Wilson, 2013). Several authors further documented the implication of travelers, including medical tourists, in the global spread of ABR (Coetzee et al., 2016; Fernandes et al., 2016; Holmes et al., 2016). The travelers likely acquired antibiotic-resistant bacteria and/or ARGs following the consumption of contaminated foods, water and/or contact with the environment in developing countries (Coetzee et al., 2016; Fernandes et al., 2016).

The variations observed between countries, with an important north-to-south gradient, reveal that the spread of antibiotic-resistant bacteria in the food chain is likely to be higher in LMICs than in developed countries (Table 2). There is clear evidence that ABR is a global public health problem, which appears to be worse in developing countries, although it threatens the world equally. Despite the fact that some governments, notably China, India, Thailand, Brazil, Malaysia, and South Africa have taken significantly actions and policies toward the rationalization of antibiotic use and containment of ABR in humans, such initiatives still lag far behind in food animals. Having good data on antibiotic consumption and trends of antibiotic-resistant bacteria and ARGs in food animals, food products and humans, as well as political commitment, are imperative to better understand and manage the concern of ABR via the food chain in developing countries.

## Prevention and containment measures of antibiotic resistance from farm-to-fork

Emergence and spread of antibiotic-resistant bacteria and ARGs in the food chain, has given rise to severe health and socio-economic repercussions globally. Resistant foodborne infections are amongst the main public health issues associated with the threat of ABR in the food chain. This global concern equally affects developed and developing countries, and may cause outbreaks and pandemic situations (Padungtod et al., 2008). The problem is more serious in the developing world, where resistant infections significantly increase morbidity and mortality rates, whereas in developed countries, these infections will enhance therapeutic costs (Harbarth et al., 2015). The majority of developing countries are not implementing adequate measures to prevent and curb the spread of ABR from farm-to-fork, thus, posing a significant threat for global public health. Combating ABR effectively at a global scale means tackling it in the developing world first, whether in the food chain or not. The One Health approach was endorsed to address the threat of ABR by supranational entities, following the coalition between the WHO, FAO, and OIE referred as the “Tripartite Alliance.” The WHO, in collaboration with its Tripartite partners, published the Global Action Plan on Antimicrobial Resistance in 2015, an attempt to counteract effectively this worldwide concern (WHO, 2015a). The FAO similarly launched its Antimicrobial Resistance Strategy in September 2016 to support the implementation of the WHO's Global Action plan in the food and agricultural sectors (FAO, 2016). Noting that ABR in the food chain is a serious global threat which is considerably neglected/under-estimated in developing countries, this section consequently proposes solutions for an effective containment of ABR from farm-to-fork, albeit relatively applicable in both developed and developing nations. The five strategic objectives of the WHO's Global Action Plan, namely (i) heighten awareness and understanding on antibiotic use and antibiotic resistance, (ii) strengthen knowledge via surveillance and research, (iii) reduce infectious diseases, (iv) optimize rational antibiotic use, and (v) mobilize resources, research and development, have been used as framework to set out integrated prevention and containment measures of ABR in the food chain (WHO, 2015a, Table 3).

**Table 3:** **Summary of prevention and containment measures of antibiotic resistance from farm-to-fork**.

**Measures**	**World Health Organization's Global Action Plan Strategic Objectives**				
	**Increase awareness**	**Strengthen surveillance**	**Reduce infectious diseases**	**Optimize rational antibiotic use**	**Mobilize resources, strengthen research and development**
Basic	National awareness campaigns on antibiotic usage and ABR	Integrated food chain surveillance systems	Implement biosecurity measures	Prohibit the growth-promotion use of antibiotics	Political will
	Food safety awareness campaigns	Establish reference laboratory		Prohibit unrestricted access of antibiotics	
	Achieve effective “culture change”	Harmonized-laboratory methods	Institute massive immunization campaigns	Establish guidelines for veterinary use of antibiotics	Sustainable commitment involving all stakeholders
		Reinforced education and fostered excellence			Assess and manage food safety risk
Moderate	Cross-disciplinary research	Research on total bacterial resistome and mobilome	Organic farming practices	Develop methods to verify judicious antibiotic use	Institute sustainable collaboration (North-to-South, South-to-South, Private-to-Publi)
	Provide assistance, support and training to occupationally exposed workers		Well-controlled extensive farming practices	Institute veterinary oversight	
			Reinforce veterinary legislation and enforcement policies		Leverage resources
Advanced	Evaluate the impact of the educational programs	State-of-the-art methods	Predictive microbiology	Institute Incentives/disincentives	Pre-, pro-, and syn-biotics
			Diagnostic tool based-nanoscale materials	Implement legal regulatory framework	Phage-related therapies
					Genetically modified food animals
					Nano-antibiotics

### Heightening awareness and understanding of antibiotic resistance

#### Public awareness campaigns on antibiotic use and antibiotic resistance

Heightening national awareness campaigns on antibiotic use, ABR, and food safety are important to address this global challenge from farm-to-fork, as knowledge leads to prevention. Well-designed and simple key messages that target populations and engage all-stakeholders (viz. farm, and abattoir workers, food handlers, consumers, veterinarians, pharmacists, food production industries, healthcare workers) are essential for successful awareness campaigns (Harbarth et al., 2015). Strengthened awareness through mass and social media continuously repeating key messages may contribute to effectively lessen antibiotic consumption and ABR rates (Harbarth et al., 2015). The choice of indicators and targets are crucial in monitoring the impact of such campaigns (Harbarth et al., 2015).

Global awareness campaigns on antibiotic use and ABR have been increasingly advocated, with sustainable communication measures having been implemented at international level, e.g., the “European antibiotic awareness day” in EU, the “get smart: know when antibiotics work” in US, and the first “World Antibiotic Awareness Week” held in November 2015. Although challenging, awareness or educational campaigns are relatively successful, with some good examples reported across the world (Harbarth et al., 2015). Farmers, abattoir workers, food handlers, and veterinarians are not the only ones affected by the threat of ABR in the food chain, but all categories of the general population must be involved and educated.

#### Food safety awareness campaigns

Food safety is a scientific discipline aiming to ensure safe food and prevent foodborne diseases throughout all stages of the food production chain including handling, transport, storage, and preparation. Food safety awareness campaigns are essential to improved consumer's and food handlers' knowledge to prevent foodborne infectious diseases and hazards (including antibiotic residues, ARGs, and antibiotic-resistant bacteria). Some basic food safety measures, such as convenient hand-washing with water and soap several times during the day (especially before and after meal preparation, after usage of toilets), effective vegetable-washing, adequate cooking temperatures, and food storage are important to reduce the spread of antibiotic-resistant bacteria and the prevalence of ABR- foodborne infections. In this regard, the WHO has summarized these practices into a simple and strong tool called “five keys to safer food” to help ensure food safety from farm-to-fork (Mwamakamba et al., 2012). It is the responsibility of stakeholders involved along the food chain, including food production industries, agricultural practitioners, food handlers, and consumers to ensure safe food using the adequate food safety measures, as depicted by the WHO, albeit governments have the central role in providing an adequate framework to implement food safety-associated health promotion programs. Several countries in Africa and Asia have implemented food safety education programs using the WHO five keys to safer foods (Prabhakar et al., 2010; Mensah et al., 2012; Mwamakamba et al., 2012).

#### Behavior change, education, and training

Behind these sensitization efforts, human behavior, whether through inter alia individualism, lack of education, culture or social beliefs, is an important factor that needs to be considered when educating/creating an awareness on antibiotic use and ABR, as new knowledge is not immediately translated into new practices. For a successful intervention, it is important to implement cross-disciplinary research involving anthropologists, sociologists, psychologists, and ethnographers to investigate the target groups' perception and knowledge beforehand. This will enable the most efficient strategies to be developed to modify behaviors, and achieve and enact effective “culture change” (Prabhakar et al., 2010; Mensah et al., 2012; Mwamakamba et al., 2012; Harbarth et al., 2015).

Reinforced education and fostering excellence in both private and public veterinarians about conservative prescription of antibiotics, the emergence of ABR and the One Health approach have a crucial role to play in containing ABR from farm-to-fork. The OIE provides guidelines on initial veterinary education, highlighting the basis for an organized profession with high-quality professionals (OIE, 2016). It is also essential to provide assistance, support and training to occupationally exposed workers (viz. to farmers, abattoir and food workers), as well as guidance on rational antibiotic use and good agricultural practices, according to their education level. Such initiative must be supported at a global level, and adapted at the local level through leadership from relevant ministers, industries, agricultural practitioners, and veterinarians. Monitoring strategies must also be developed to evaluate the impact of the educational programs.

### Strengthening surveillance and research

#### Integrated food chain surveillance systems

An essential step toward addressing the public health threat of ABR is to determine the resistance burden from different ecological niches in the entire food chain (farm, abattoir, market, etc.). This is more evident in developing countries, where data on ABR, antibiotic use and foodborne illnesses is scarce. Integrated food chain surveillance systems, conducting sustainable research (long-term epidemiological and molecular studies) from farm-to-fork, endorsing the One Health approach and the Codex Alimentarius Commission guidelines, and reporting a combination of passive, active, and outbreak data sources are needed to fill the data gaps, as well as to assess the impact of public awareness campaigns on rational antibiotic use and ABR (Prabhakar et al., 2010; FAO and WHO, 2011; Acar and Moulin, 2013). The generated information from such surveillance could inform decision-makers, evidence-based policies, and help to allocate appropriate resources for preventing and containing ABR via the food chain. These systems further need to establish harmonized laboratory methods and provide specifications for the target populations, samples, settings and bacteria, in keeping with the WHO's AGISAR, in order to compare results nationally and internationally.

The NARMS, DANMAP, and MARAN are well-known integrated food chain surveillance systems that have been established in developed countries to undertake research along the food chain, combine data from various sources and use strong multi-sectorial partnerships with other entities (DANMAP, 2014; FDA, 2014; NethMap-MARAN, 2015). Colombia is a notable developing country example, having successfully piloted an integrated surveillance system to monitor trends in antibiotic resistance on poultry farms, abattoirs and retail markets in order to develop adequate ABR prevention and containment measures (Donado-Godoy et al., 2015). The pilot Colombian Integrated Program for Antimicrobial Resistance Surveillance (COIPARS) was able to meet animal health and welfare requests of food producers, and address public health concerns associated with the antibiotic use in food animals and ensure food safety. The COIPARS is regarded as a model in Latin America by the International Molecular Subtyping Network for Foodborne Disease Surveillance (PulseNet International) and the WHO Global Foodborne Infections Network (Donado-Godoy et al., 2015). The WHO AGISAR has further used COIPARS as a guide to harmonize methods for monitoring of ABR and antibiotic use in food animals in the Americas (Donado-Godoy et al., 2015).

Similarly, Mexico implemented a pilot on integrated food chain surveillance system to prevent and contain *Salmonella* spp., *Campylobacter* spp., and *E. coli* from farm-to-fork in four regions and ascertained its cost-effectiveness. Samples were collected from sick and healthy humans, locally produced retail chicken, pork, beef, and their intestines at slaughterhouses, with a sample size representative of the regional consumption of each retail meat using standardized laboratory protocols (Zaidi et al., 2008, 2015; Donado-Godoy et al., 2015). Within a 3 years-period, and with a limited financial investment, the Mexican's integrated food chain surveillance system was able to yield meaningful epidemiological data on emerging public health threats, identified effectively strains of greatest public health threat, as well as the main food animal reservoirs, showing that these systems are technically and economically feasible in developing countries (Zaidi et al., 2008, 2012, 2015).

#### Reference laboratory and harmonized-laboratory methods

National reference laboratories are essential to provide harmonized, high quality, and precise information when assessing ABR bacteria (indicator, foodborne, and emerging bacteria) with the potential to threaten food safety. The majority of developed countries have well-designed reference laboratories that focus their activities on humans as well as animals, while few LMICs have established national reference laboratories, with those that do mainly focusing on humans. National reference laboratories in which bacteria are tested for antibiotic susceptibility exist in 6 out 47 and 6 out of 21 countries of the WHO African and Eastern Mediterranean Regions respectively (WHO, 2015b). Reorientation of the research strategy to include food animals, food derived products, and humans is vital to contain the spread of ABR from farm-to-fork in developing countries (de Balogh et al., 2013).

#### Research on the total bacterial resistome

Given that food animals are key and persistent reservoirs of ABR, basic food chain surveillance programs focus primarily on foodborne or indicator bacteria. Effectively curbing the emergence and spread of ABR needs to consider the role of commensal flora in the emergence of resistance. Antibiotic use affects and selects largely resistant bacteria in commensal flora, which may subsequently transmit their ARGs to the pathogenic ones (Andremont, 2003). The commensal genetic pool is so huge that it involves several pathways of transferring resistance, including mutations and complex resistance mechanisms (HGT) that are either expressed or silent within subdominant species, and are able to move freely between species (Andremont, 2003). Assessing the whole bacterial resistome and mobilome through food surveillance systems will enable the detection of emerging antibiotic-resistant bacteria and ARGs from farm-to-fork. This is important to inform issues that are often ignored by basic food chain surveillance programs, these being to detect early outbreaks and undertake timely correct actions. This is obviously more feasible in developed and well-resourced countries.

#### State-of-the-art methods

Detecting antibiotic-resistant bacteria and ARGs is based on culturing, antimicrobial susceptibility testing (AST), and polymerase chain reaction (PCR). Although AST and PCR-based methods are excellent for phenotyping resistance profiling and detecting ARGs respectively, both require bacterial culture and do not enable the discovery of distantly related or unknown elements that may be present, and which are particularly crucial in the farm-to-fork continuum. Whole genome sequencing enables the detection and exploration of complete bacterial genome, with a view to identify new genetic traits. Metagenomics also allows the analysis of the whole genome and identification of unknown genetic elements, regardless of the cultural characteristics of the bacteria (Allen, 2014; Thanner et al., 2016). Single nucleotide polymorphisms, novel resistance genes and unforeseen aspects of ARG ecology may be identified through the use of whole genome sequencing and functional metagenomics, respectively (Allen, 2014; Thanner et al., 2016). Plasmid sequence analysis and reconstruction may also be performed using whole genome sequencing and metagenomics. Microarray expression analysis provide useful insights into expressing profiles of bacteria under antibiotic pressure, while recent transcriptomics and meta-transcriptomics tools identifying functional genes are also an important advancement in detecting new genetic elements (Thanner et al., 2016). State-of-the-art tools may be very useful to understand transmission dynamics of resistance gene expression through the different ecological niches prevailing in the food chain (Thanner et al., 2016). Again, these are more feasible in developed and well-resourced countries.

### Reducing the burden of infectious diseases

#### Immunization and biosecurity

Unprecedented global food demands result in farmers' reliance on antibiotics to produce large quantities of animal protein at low cost although. Evidence exits that improving animal welfare and health have the potential to diminish this over-dependence on antibiotics without affecting productivity and costs. The use of vaccines is recommended as method of preventing infections in animals and should significantly reduce antibiotic consumption while increasing the productivity. Although already available against some viral, parasitical, and bacterial infections (Table 4) in food animals, their routine use is generally limited due to few incentives promoting them and the absence of disincentives prohibiting antibiotic consumption in the food production industry (Woolhouse et al., 2015). Mass vaccination campaigns should be implemented as in humans to ensure animal health, as the greater the use of vaccines, the lesser the incidence of infections (Woolhouse et al., 2015). Vaccines can effectively protect animals against bacterial infections, but may require regular “updates” to ensure their continuous efficacy (O'Neill, 2015b).

**Table 4:** **List of some available veterinary bacterial vaccines**.

**Target animal**	**Target pathogen**	**Brand name**	**Characteristics**	**References**
Pig	*Lawsonia intracellularis*	Enterisol Ileitis	Live oral vaccine	Guedes and Gebhart, 2003
Fish	*Yersinia ruckeri*	AquaVac ERM	Killed oral vaccine	Meeusen et al., 2007
Fish	*Aeromonas salmonicida*	AquaVac Furuvac	Killed oral vaccine	
Fish	*Vibrio anguillarum*	AquaVac Vibrio	Killed oral vaccine	
Chicken	*Salmonella spp*.	Megan Vac1 MeganEgg	Live vaccine	Babu et al., 2004
Cattle	*Brucella abortus*	RB-51	Rifampin-resistant mutant	Moriyon et al., 2004
Pig	*Actinobacillus pleuropneumoniae*	PleuroStar APP	Recombinant proteins	Van Overbeke et al., 2001
Chicken	*Mycoplasma gallisepticum*	Vaxsafe MG	Live vaccine for eye drop administration	Barbour et al., 2000
Chicken	*Mycoplasma synoviae*	Vaxsafe MS	Live vaccine for eye drop administration	Meeusen et al., 2007
Turkeys	*Bordetella avium*	Art Vax	Live for spray inhalation or drinking water	
Sheep	*Chlamydophila abortus*	Ovilis Enzovax	Live vaccine for intramuscular or sub-cutaneous injection	
Pig	*Actinobacillus pleuropneumoniae*	Porcilis APP	Outer membrane proteins	

Biosecurity measures in food and agriculture should further significantly reduce or eliminate ABR-bacteria from farm-to-fork, and thereby, lower the burden of infectious diseases. Biosecurity refers to a holistic concept used to define a set of ongoing measures taken to reduce the risk of the emergence/introduction and dissemination of diseases at herd-, region-, and country-levels (FAO, 2003; FAO and WHO, 2011). It uses “One Health” as a framework, and benefits from this integrated approach by encompassing various concepts, such as good agricultural practices, good hygiene practices, good veterinary practices, hazard analysis, and critical control points (HACCP)-based procedures and microbiological risk assessment and management (FAO, 2003; FAO and WHO, 2011). When animals are healthy, antibiotics are not needed to treat them. However, when ABR occurs, and effective barriers (e.g., good agricultural practices, good hygiene practices, and HACCP-based measures) are present, subsequent transmission will be impeded, with food safety, food security, and public health being ensured. Biosecurity should then play a fundamental role in any disease control program as it expands beyond agricultural production to human and environmental health (Nahar et al., 2014; Postma et al., 2016).

#### Farming practices

Well-controlled extensive farming practices using small chemical substances and that favor animal health and welfare to limit the development of infectious diseases and reduce antibiotic use (e.g., all-in-all-out, extensive free-range systems), should be promoted and implemented rather than intensive farming practices. Organic farm systems have recently received considerable interest to meet the global food demand in an effort to reduce the extensive use of antibiotics (Prabhakar et al., 2010). In comparing the occurrence of ABR in food animals in conventional and organic farms, Österberg et al. (2016) reported a significant lower prevalence of ABR-*E. coli* isolated from organic pig farms than from conventional ones in four European countries (Österberg et al., 2016). This suggests that organic farms could be valuable agricultural alternatives to reduce the burden of ABR, although further research is needed to ascertain the consequences related to such practices and improve the productivity. Sweden, the first country that implemented a total ban of antibiotic growth-promoters in food production industry in 1986, is one of the world's references for containment of ABR in the food chain through well-implemented and adequate farming practices (O'Neill, 2015b). Following the 1986's ban, to facilitate the passage to new farming practices, Swedish authorities developed guidelines on management, hygiene, medication, and feed to improve animal health and prevent infectious diseases. Swedish efforts were considerably focused on problem-oriented research and provided extensive support for farmers (O'Neill, 2015b). This led to decrease of sales of antibiotics used in food animals from 45 tons to around 15 tons of active substances by 2009 (O'Neill, 2015b). In contrast, up to a third of the projected 67% global increase in antibiotic consumption in food animals will be attributable to shifting agricultural practices in LMICs, where extensive farming systems will be substituted by large-scale intensive farming (Van Boeckel et al., 2015). This suggests that LMICs should follow the Swedish experience for sustainable agriculture independent of antibiotics although data relative to such initiatives was limited.

#### Rapid infection diagnostics

There are significant advantages of rapid diagnostic tools, such as culturing, PCR, microarray, and whole genome sequencing, being more readily available to quickly identify bacterial infections, resistance patterns, and determine appropriate treatment in animals and humans (Woolhouse et al., 2015). Chromogenic tests and Matrix-assisted laser desorption/ionization time-of-flight have been proposed, as they allow not only rapid bacterial identification and but also effective resistance profiling. These tools may be suitable for LMICs where diagnostic tool must be relatively economical, rapid, easy to use, and requires less logistic infrastructure (Holmes et al., 2016).

Several mathematical models, such as Predictive Microbial Modeling Lab (PMM-Lab), Pathogen Modeling Program (PMP), Geeraerd and Van Impe Inactivation Model Fitting Tool (GInaFiT), and Combined dataBase (ComBase) Predictor, that predict the bacterial behaviors in various stages of production (e.g., processing, storage, and distribution) have recently received significant interest in the food production industry (Plaza-Rodríguez et al., 2015). Predictive microbiology generates substantive quantitative data and useful estimates that can assist in decision-making during food production process, HACCP-based procedures and food safety risk analysis and management (Plaza-Rodríguez et al., 2015; Thanner et al., 2016). Predictive microbiology could be valuable to reduce antibiotic use and infectious diseases, and contain ABR. However, it must be optimized to encompass important factors, such as the total bacterial resistome, microbial loads, biological mechanisms, and cross-species transmission, with outcomes not only being lethality, but also incidence of diseases, level of pathogenicity and prevalence of antibiotic-resistant bacteria and ARGs (Plaza-Rodríguez et al., 2015; Thanner et al., 2016).

The Artificial Neural Network (ANN) is an advanced mathematical model capable of performing large and simultaneous computations for data processing and knowledge representation. It is able to combine data of different nature collected from various sources, populations and protocols, thereby forming an integrated diagnostic system. Several studies have demonstrated that ANNs could be built and trained to recognize bacterial resistance patterns, and to generate adequate conclusion in terms of ABR profiles (Budak and Übeyli, 2011; Lechowicz et al., 2013). Budak and Übeyli (2011) used ANN to predict the resistance profiles of *Salmonella spp*. to Ampicillin, Chloramphenicol, and Trimethoprim-sulfamethoxazole, and reported overall accuracies of more than 95% (Budak and Übeyli, 2011).

Modern methods, such as Fourier Transform Infrared Spectroscopy (FTIR) and Attenuated Total Reflection-based (ATR)-FTIR, that detect the presence and measure the infrared spectra of intact microbial cells have also been proposed for rapid bacterial strains detection in the food production chain (Rebuffo-Scheer et al., 2007; Lechowicz et al., 2013). The combination of infrared spectroscopy with ANN also allows the rapid identification of bacteria and determination of resistance patterns. Lechowicz et al. (2013) demonstrated that the Multi-Layer Perception network built using the ANN-based FTIR method was able to successfully detect uro-pathogenic *E. coli*, and to classify strains susceptible and resistant to cephalothin with more than 90% of accuracy. Although these tools require sufficient skills, they present an adequate level of accuracies to differentiate bacteria and determine resistance patterns, and could be used routinely for laboratory diagnostics (Rebuffo-Scheer et al., 2007; Lechowicz et al., 2013).

Nanoscale materials have also emerged as an innovative approach to enable rapid, sensitive and cost-effective diagnosis, and to adequately determine bacterial resistance profiles. Antibody-conjugated nanoparticles have reported to be able to identify highly pathogenic *E. coli* O157:H7 within 20 min. A rapid diagnostic tool, using gold nano-wire arrays in conjunction with a linker arm coated to specific *E. coli* antibodies, has recently been developed to detect urinary tract infections (Huh and Kwon, 2011). On-going research using magnetic nanoparticles, such as dextrancoated super-magnetic iron oxide nanoparticles attached with con-A conjugated nano-sensors, is assessing bacterial metabolic activity and antibiotic susceptibility in opaque media, including blood and milk, without any sample preparation. The intention is to achieve fast and reliable diagnostic assays for bacterial infections adapted to the food production chain, although this is not as yet commercially available (Huh and Kwon, 2011).

Rapid diagnostics will allow agricultural practitioners to identify early infection in animals, to separate those that are infected from others, prevent the spread of infection to the whole herd or flock, and thereby reduce antibiotic use (FAO, 2015; O'Neill, 2015b). Technical and financial barriers, as well as resistance to the adoption of innovative ideas are slowing the implementation of such measures around the world, particularly in developing countries.

### Optimizing rational antibiotic use

#### Ban on antibiotic use

The burden of ABR has reached and surpassed the pace of development of new antibacterial agents entering clinical use, and has led to a radical re-evaluation of antibiotic use. Given the growing global concern about the spread of antibiotic-resistant bacteria from animals to humans, there is a significant interest in phasing out antibiotics in the food production industry. LMICs should determine their annual antibiotic consumption in food animals and prohibit the growth-promotion use of any antibiotic substances which the WHO considers to be “critically, highly and important antimicrobials in human medicine,” as well as those not currently approved for veterinary use (WHO, 2011; OIE, 2015; O'Neill, 2015b). Avoparcin, was the first antibiotic growth-promoter banned in 1995 in Denmark, followed by virginiamycin in 1998 and a complete ban of antibiotics in 2000 (DANMAP, 2014). The effects of the ban were noticeable as the country experienced significant reduction in antibiotic use and ABR, without affecting the pig and poultry productions which continued to thrive albeit with a small drop in productivity at the inception of the ban and until biosecurity efforts were enhanced. Danish farmers increased their pig production by 47% over the period 1992–2008, preserving the standing of country as among the greatest exporters of pork worldwide, with 90% of their pig production being exported (O'Neill, 2015b). Denmark thus illustrated that very productive and sustainable food production is possible alongside low antibiotic use (O'Neill, 2015b). The majority of developing nations are still far behind a total ban of antibiotics for growth-promotion purposes. Considerable efforts are thus needed by way of an incremental process encompassing cultural, political, and socio-economic contexts while taking cognizance of the urgency and gravity of the ABR concern.

#### Prohibit unrestricted access of antibiotics

As ARGs are generally carried on mobile genetic elements and all antibiotic families have the potential to select, to a certain degree, cross-resistance to other families. The unrestricted access of antibiotics to farmers should thus be prohibited unless under veterinary prescription or oversight. Meaningful veterinary oversight should further be mandatory when classes of antibiotics currently approved for dual use (both animal and human health) to treat or prevent infectious diseases are prescribed. It is also important to develop methods to verify judicious antibiotic use, establish goals for measuring progress and guidelines for consuming veterinary antibiotics (O'Neill, 2015b; OIE, 2016). In 2009, Dutch government implemented a registration process for veterinary prescription of antibiotics. As a result, between 2007 and 2012, Dutch antibiotic sales in food animals decreased by 56% without any loss in terms of productivity and profits. Dutch farmers have changed their farming practices from over-reliance on antibiotics to improved animal health and welfare (O'Neill, 2015b). To the best of our knowledge, such measures have not been significantly implemented in food animals and aquaculture in LMICs, where regulatory framework and legislation implementation are further limited.

#### Legislative regulation and enforcement policies

The OIE recommended reinforcing veterinary legislation and enforcement policies to ensure compliance with the law and regulations promoting responsible and prudent use of Veterinary Critically Important Antimicrobial Agents (VCIA), Veterinary Highly Important Antimicrobial Agents (VHIA), and Veterinary Important Antimicrobial Agents (VIA) (OIE, 2015, 2016). When antibiotics are justifiably required, it is essential to follow the established principles and criteria for antibiotic use, according to the OIE “List of Antimicrobial Agents of Veterinary Importance.” Achieving a balance between minimizing antibiotic use in food production industry, while meeting the unprecedented rise of worldwide food demands, is a fundamental food security/food safety challenge that must be considered when defining target goals for reducing antibiotic use. It is therefore important that every country involves stakeholders from various sectors (governments, industries, experts, practitioners, and international entities) to set an ambitious but realistic and achievable targets to reduce antibiotic consumption (O'Neill, 2016). Developing countries should examine and adapt the Danish, Dutch, and Swedish experiences as well as acquire scientific expertise from these countries in order to reduce their antibiotic consumption in food animals and aquaculture while preserving their productivity. It is further essential to promote and ensure sustainable agriculture not over-reliant on antibiotics in the developing world to reach the United Nations Sustainable Development Goals by 2030. Sustainable support from developed countries, funding agencies and international entities is essential to reduce antibiotic use and ABR in food animals and aquaculture in LMICs.

### Sustainable investment and development of new medicines

#### Political will

The human and economic burden associated with containing ABR are trivial compared to the cost of complacency or inaction. ABR will account for more than 10 million deaths by 2050, with a cumulative global economic loss of US $100 trillion over the 35 next years if not addressed substantively (O'Neill, 2015a). ABR should be considered a national priority to retain the attention, ensure sustainable investment and allocate resources to successfully contain it. Governments should translate surveillance estimates into policies leading to implementing long-term interventions and activities in animal husbandry systems and agriculture to ensure animal welfare and health, and to contain ABR at local, national, regional, and international levels. More public and private partnerships are also needed to assess the problem in its broader context and find lasting solutions. Active political will is thus indispensable to enact sustainable engagement, investment, research, and alternatives to antibiotics in the food production industry (O'Neill, 2015b; Woolhouse et al., 2015).

#### Sustainable engagement

The need to address and fill the gaps in ABR knowledge have never been more imperative. Sustainable engagement of inter alia policy makers, agricultural practitioners, veterinarians, antibiotic providers, industries, food suppliers, the public, and others who may contribute to long-term solutions is an essential component to effectively contain ABR via the food chain. Multi-sectorial and coordinated efforts should cohesively identify and target research priorities as well as increase research funding to effectively overcome this global challenge (Woolhouse et al., 2015; O'Neill, 2016).

#### Leverage resources

Addressing the issue of ABR globally requires greater efforts in developing countries, where interventions may be restricted as a result of inadequate resources. It is thus important to approach to donor funding agencies and supranational entities to improve prevention and containment of ABR via the food chain. An example is the Fleming Funds with its £265 million allocated by the UK government to help improve surveillance in LMICs (O'Neill, 2016). The World Bank's International Development Association (IDA) is another funding mechanism through which LMICs could request support to strengthen surveillance programs, built laboratory-capacity, and prevent and contain ABR. More international support to LMICs are however still needed.

#### Alternatives to antibiotics for food animals

The global scientific community recognizes the urgent need to reinvigorate the therapeutic pipeline with new antibiotics. The community is further aware of the need for effective and adequate alternative solutions to antibiotics in food animals as an essential strategy to circumvent the challenge of ABR. The range of these potential alternatives is, for the most part, similar to those already documented in human medicine and may include prebiotics, probiotics, synbiotics (viz. mixing of prebiotics and probiotics), substances targeting bacterial communication (quorum sensing), phage enzymes, phage-related therapies and the use of genetically modified food animals (already resistant to infection; Woolhouse et al., 2015).

Synthetic biology (SB) provides a novel approach to discover new antibiotics, and to exploit and improve existing drugs. It uses the genomic data generated by state-of-art technology to identify biological machineries of various bacteria predicted to be able to produce antibiotics. Synthetic bacteria with beneficial functionalities are designed and new antibiotics bio-synthesized with novel antibacterial activity under artificial and engineered control systems (Takano and Breitling, 2014). SB offers an exciting prospect to expand the therapeutic arsenal, as it could generate libraries of novel, predicted and different drug variants with broader activities and better pharmacokinetics (Takano and Breitling, 2014).

Nano-antibiotics, either having antimicrobial activity by themselves or as novel nano-sized drug delivery platforms, are also part of the most interesting alternatives to antibiotics. Unlike other antibiotic substances and proposed strategies, nano-antibiotics exert their activity through several pathways, such as (i) a combination of numerous mechanisms to inhibit bacteria (e.g., nitric oxide-releasing nanoparticles, chitosan-, and metal-containing nanoparticles) (ii) encapsulating several antibiotics within the same nanoparticles, (iii) reduced uptake and increased drug efflux, and (iv) site specific action releasing high concentration of antibiotics at the site of infection (Huh and Kwon, 2011; Pelgrift and Friedman, 2013). Nano-antibiotics offer several advantages, not only in overcoming ABR and reducing acute adverse effects (although toxicity upon long-term exposure is yet uncertain) but they also remain stable on long-term storage and are cost-effective preparations compared with antibiotic synthesis (Huh and Kwon, 2011; Pelgrift and Friedman, 2013). Considerable investments in research are still needed before any of the above possibilities become commercially available on a global scale and can effectively replace antibiotics.

## Conclusion

ABR is a global public health challenge with severe health and socio-economics repercussions that is significantly influenced by antibiotic use in food animals. Combating ABR effectively at a global scale means addressing it equally in the developed and developing world. Multifaceted, comprehensive and integrated strategies, as advocated by the WHO Global Action Plan and FAO Action Plan in line with the One Health approach, are urgently required to (i) prevent the transmission of ABR and infectious diseases from farm-to-fork, (ii) circumvent potential pandemic situations, and (iii) preserve the efficacy of antibiotics and assure food safety, food security, and global health. Countries should therefore follow WHO, OIE, and FAO recommendations to implement national action plans encompassing human, (food) animal, and environmental sectors to improve policies, interventions and activities that address the prevention and containment of ABR from farm-to-fork.

## Author contributions

LF co-conceptualized the study, searched the literature, extracted and collated data, and drafted the manuscript. RF extracted and collated data and contribute to the writing of the manuscript. SE co-conceptualized the study, provided substantial revision of the manuscript. All authors read and approved the final manuscript.

## Funding

This work was supported by the Antimicrobial Research Unit (ARU) and College of Health Sciences (CHS) of the University of KwaZulu-Natal.

### Conflict of interest statement

SE is a member of the Global Respiratory Infection Partnership and Global Analgesic Steering Committee sponsored by Reckitt and Benckiser. The other authors declare that the research was conducted in the absence of any commercial or financial relationships that could be construed as a potential conflict of interest.
